# Supplementary material for: Identification of DLK1, a Notch ligand, as an immunotherapeutic target and regulator of tumor cell plasticity and chemoresistance in adrenocortical carcinoma
Source: bioRxiv. 2024 Oct 11:2024.10.09.617077. Preprint. [Version 1] doi: 10.1101/2024.10.09.617077 (PMC11482787; doi:10.1101/2024.10.09.617077)
Supplement: Supplement 1 [file media-1.pdf]

Supplementary Figure 1

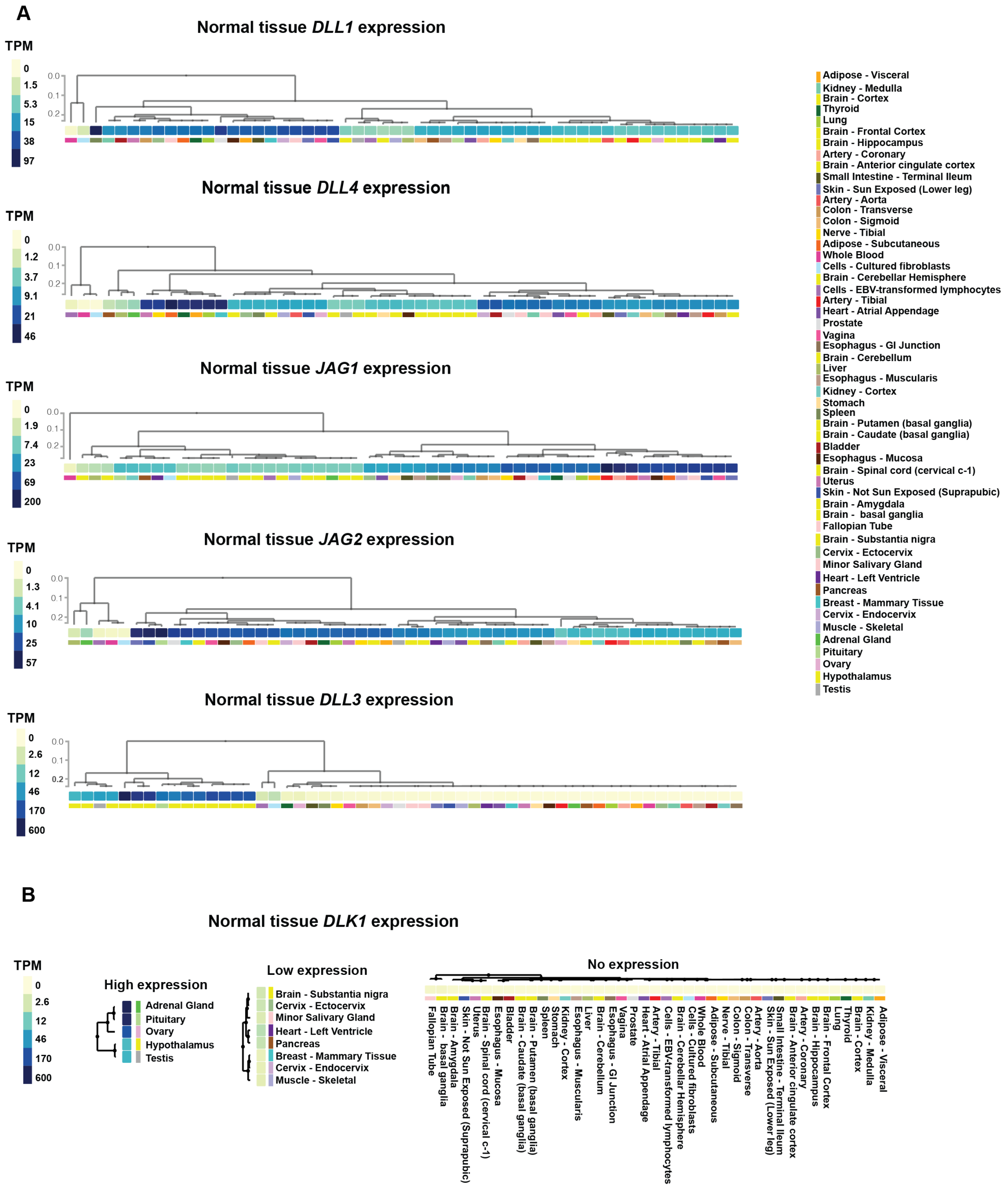

Supplementary Figure 1. Normal tissue expression of Notch ligands. (A) Normal tissue expression of *DLL1*, *DLL4*, *JAG1*, *JAG2*, and *DLL3* in the GTEx database. (**B**) Normal tissue expression of *DLK1* in the GTEx database**.**

Supplementary Figure 2

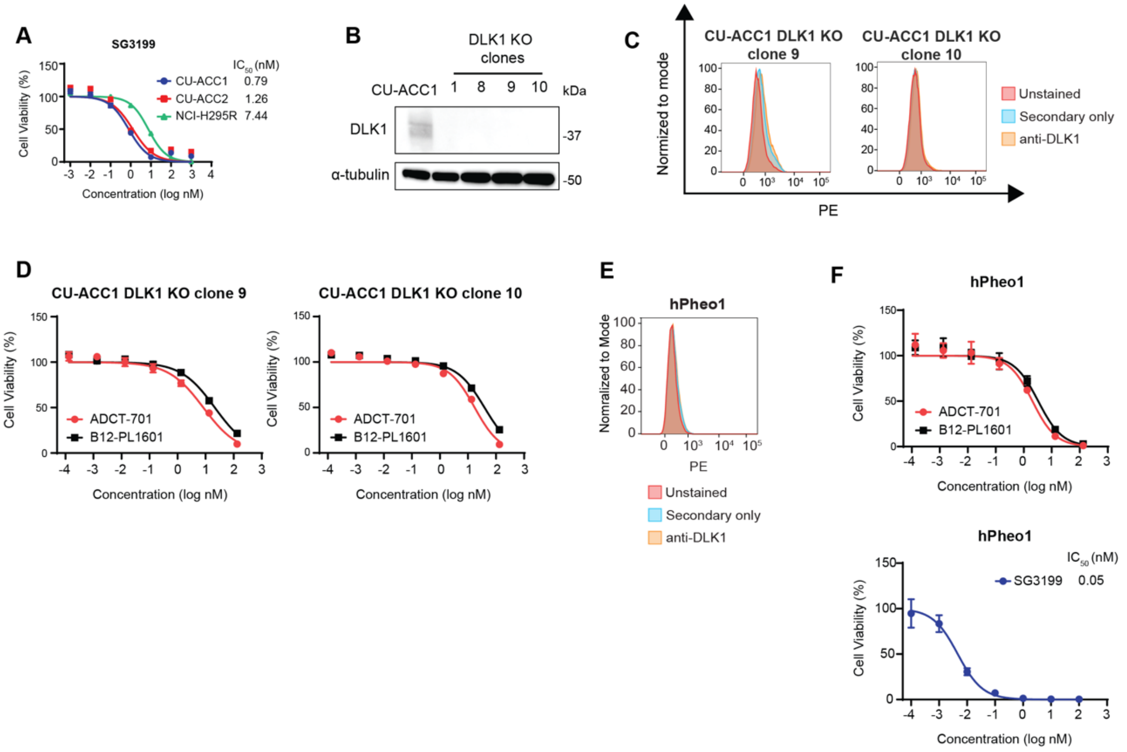

Supplementary Figure 2. ADCT-701 cytotoxicity is DLK1 dependent. (A) SG3199 cytotoxicity in CU-ACC1, CU-ACC2, and H295R cells. Cells were treated with SG3199 for 3 days. Each point represents the mean±SEM. **(B)** Immunoblot analysis of DLK1 and loading control (α-tubulin) proteins in CU-ACC1 cells with and without DLK1 KO. Four single-cell KO clones are shown. **(C)** Cell surface expression of DLK1 in two DLK1 KO clones. **(D)** Cytotoxic activity of ADCT-701 in two DLK1 KO clones. **(E)** Cell surface expression of DLK1 by flow cytometry in hPheo1 cell line. **(F)** Cytotoxic activity of ADCT-701 and SG3199 in hPheo1 cell line.

Supplementary Figure 3

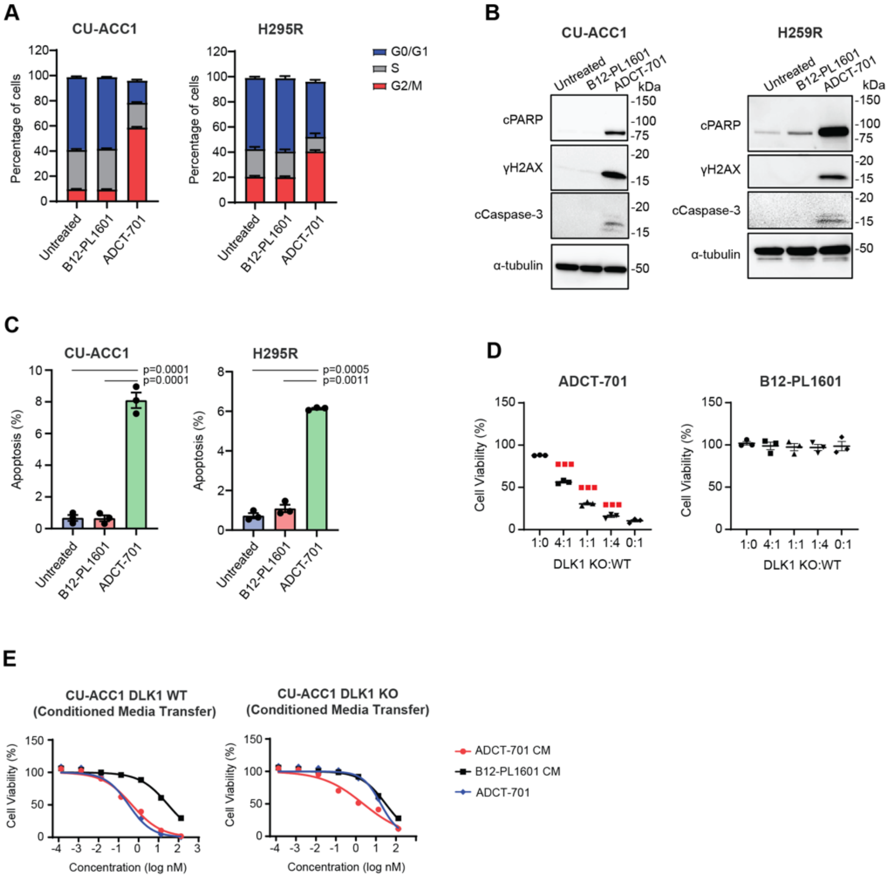

Supplementary Figure 3. ADCT-701 induces cytotoxicity in DLK1^{+} ACC cell lines through apoptosis and bystander killing. (**A**) Quantification of the cell cycle in CU-ACC1 and H295R cells treated with 0.02 µg/mL ADCT-701 or B12-PL1601 for 2 or 5 days, respectively. Data are expressed as mean±SEM (n=3 biological replicates). (**B**) Immunoblots showing DNA damage (γH2AX) and apoptosis (cleaved PARP and cleaved caspase-3) in CU-ACC1 and H295R cells treated with 20 µg/mL ADCT-701 or B12-PL1601 for 1 or 3 days, respectively. (**C**) Annexin V apoptosis assay of CU-ACC1 or H295R cells treated with 20 µg/mL ADCT-701 or B12-PL1601 after 1 or 3 days of treatment, respectively. Results represent mean±SEM (n=3 biological replicates). (**D**) Cell viability among different co-culture ratios of CU-ACC1 DLK1 KO:CU-ACC1 treated with 1 µg/mL of ADCT-701 (left) or B12-PL1601 (right) for 4 days. Red lines indicate expected viability with no bystander killing effect. Data are presented as the mean±SEM (n=3 independent experiments). (**E**) CU-ACC1 cells were treated with ADCT-701 or B12-PL1601 for 5 days. Conditioned media was collected and transferred to either CU-ACC1 or CU-ACC1 DLK1 KO cells for 5 days and cell viability was measured. Results represent mean±SEM from 3 independent experiments.

Supplementary Figure 4

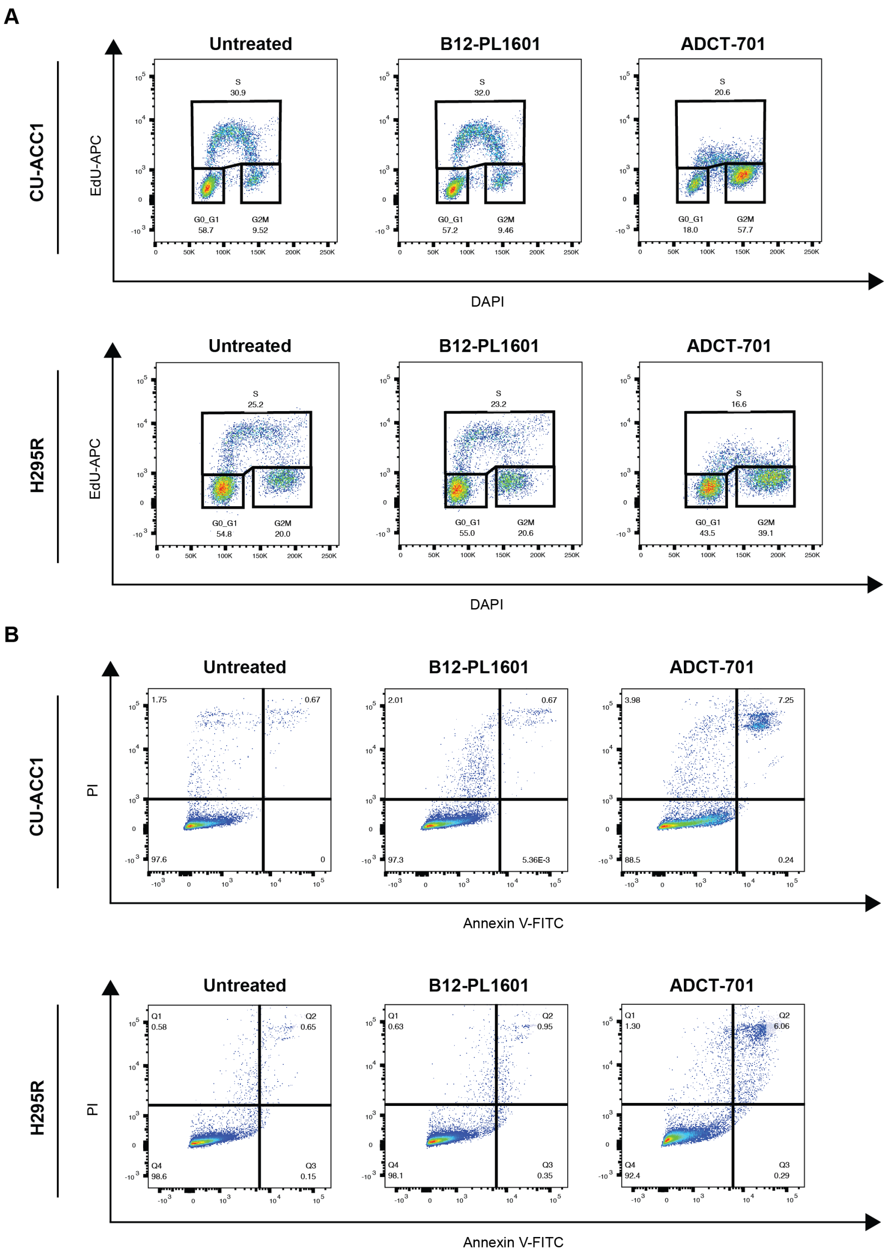

Supplementary Figure 4. ADCT-701 induces cell cycle arrest and apoptosis among DLK1⁺ ACC cell lines. (A) Representative flow graphs of cell cycle analysis show cells in S-phase (EdU+) and other phases (G1, and G2M) in CU-ACC1 or H295R cells treated with 0.02 µg/mL ADCT-701 or B12-PL1601 for 2 or 5 days respectively. **(B)** Assessment of apoptosis by flow cytometry of Annexin V and PI double positive CU-ACC1 or H295R cells treated with 20 µg/mL ADCT-701 or B12-PL1601 for 1 or 3 days, respectively.

Supplementary Figure 5

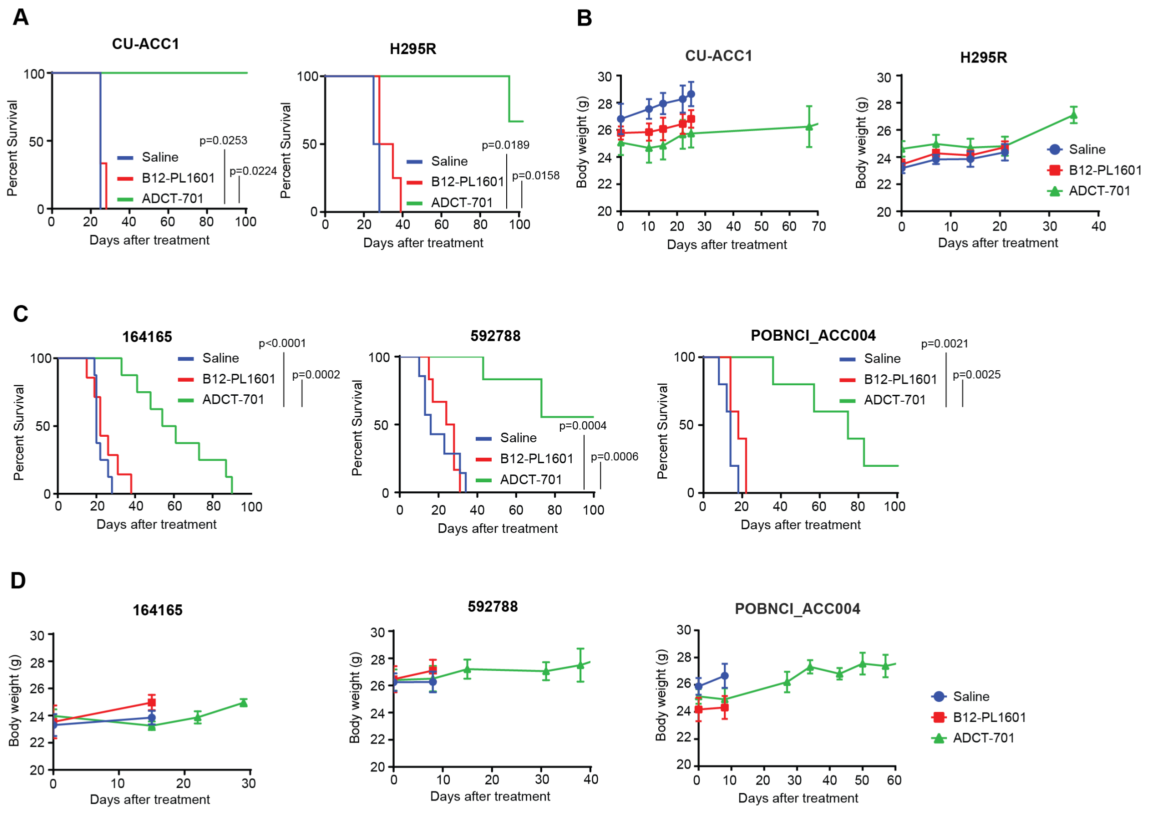

Supplementary Figure 5. ADCT-701 survival and toxicity in ACC cell-line and patient-derived xenografts. (A) CU-ACC1 and H295R xenograft survival in mice treated with normal saline, B12-PL1601, or ADCT-701. **(B)** Body weight curves with saline, B12-PL1601, or ADCT-701 treatment in CU-ACC1 and H295R xenografts. (**C**) ACC PDXs 164165, 592788, and POBNCI_ACC004 survival after treatment with saline, B12-PL1601 or ADCT-701. **(D)** Body weight curves with saline, B12-PL1601, or ADCT-701 treatment in 164145, 592788, and POBNCI_ACC004 PDX models.

Supplementary Figure 6

A

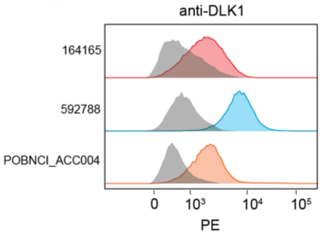

PDX	DLK1 molecules per cell
164165	3651
592788	6659
POBNCI_ACC004	3388

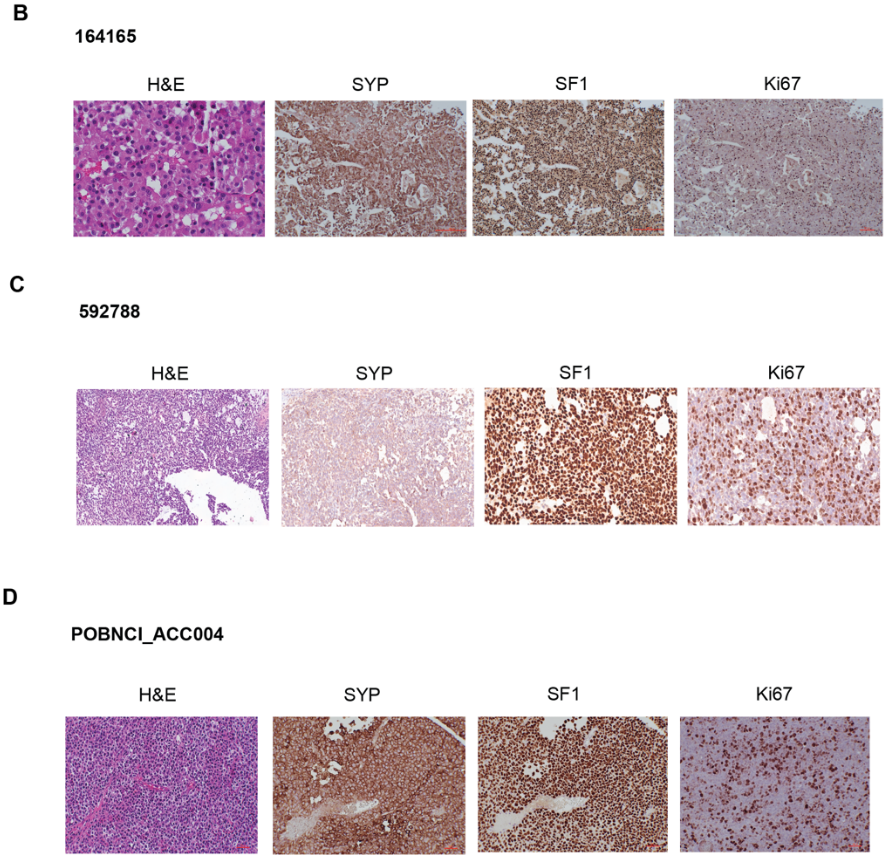

Supplementary Figure 6. DLK1 flow cytometry and validation of ACC patient-derived xenograft models. (A) Cell surface expression of DLK1 and the number of DLK1 molecules per cell among 3 ACC PDX models. H&E staining and immunohistochemistry for NE marker (SYP), adrenal specific marker (SF1) and proliferation marker (Ki67) in **(B)** 164165 PDX, **(C)** 592788 PDX, and **(D)** POBNCI_ACC004 PDX.

Supplementary Figure 7

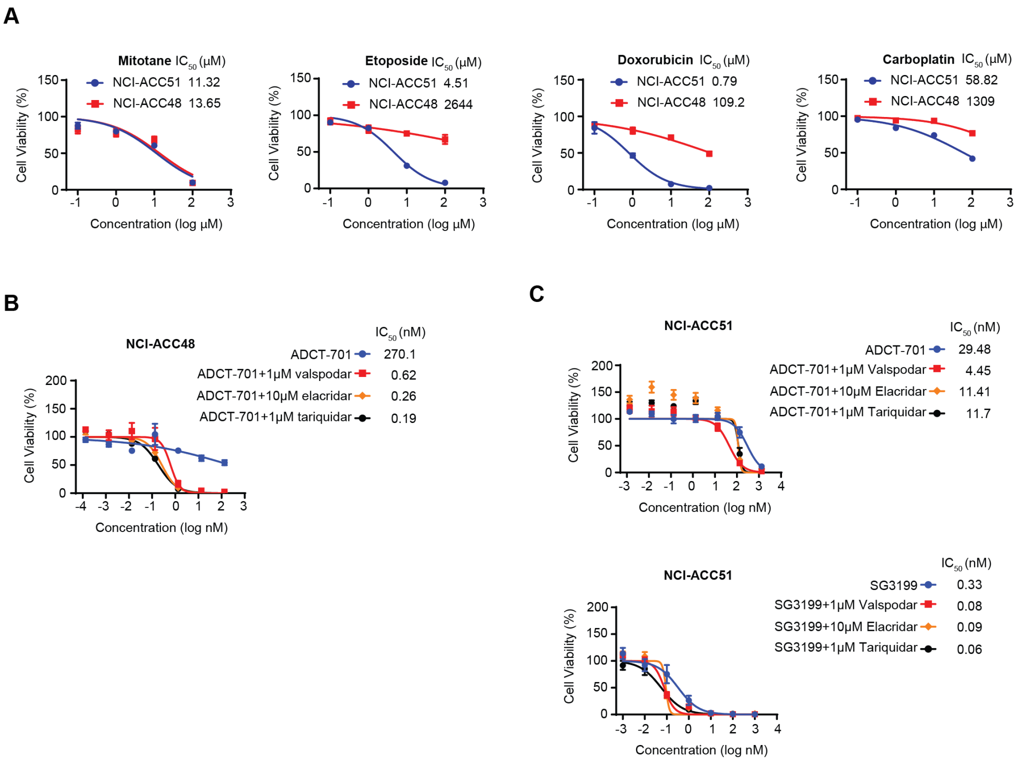

Supplementary Figure 7. ABCB1 regulates ADCT-701 and chemotherapy resistance among ACC patient-derived organoids. (A) Cytotoxic activity of mitotane, etoposide, doxorubicin, and carboplatin in two ACC patient-derived organoids (NCI-ACC48 and NCI-ACC51). Cells were treated with chemotherapeutic agents as shown for 3 days. **(B)** ADCT-701 cytotoxicity in the NCI-ACC48 patient-derived organoid with or without ABCB1 inhibitors. **(C)** ADCT-701 and **(C)** SG3199 cytotoxicity in the NCI-ACC51 patient-derived organoid with or without ABCB1 inhibitors. Cells were treated with ADCT-701 or SG3199 combined with or without ABCB1 inhibitors for 7 or 3 days respectively.

Supplementary Figure 8

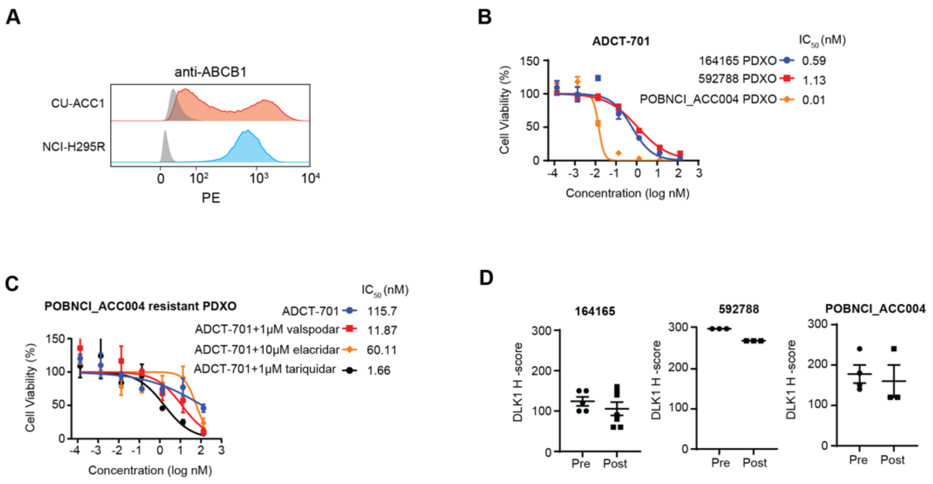

Supplementary Figure 8. ADCT-701 cytotoxicity in ACC PDX-derived organoids with or without ABCB1 inhibitors. (A) Flow cytometry histograms of ABCB1 in CU-ACC1 and NCI-H295R cell lines. Shaded gray histograms represent unstained controls for each condition. **(B)** ADCT-701 *in vitro* cytotoxicity in 592788 and POBNCI_ACC004 PDX-derived organoids. (**C**) ADCT-701 cytotoxicity in ADCT-701 resistant PDX-derived organoid treated with or without ABCB1 inhibitors. For panels **B** and **C**, cells were treated with ADCT-701 for 7 days. Each point represents the mean±SEM. (**D**) DLK1 immunohistochemistry in PDX tumors prior to ADCT-701 treatment (Pre) and after resistance to ADCT-701 (Post).

Supplementary Figure 9

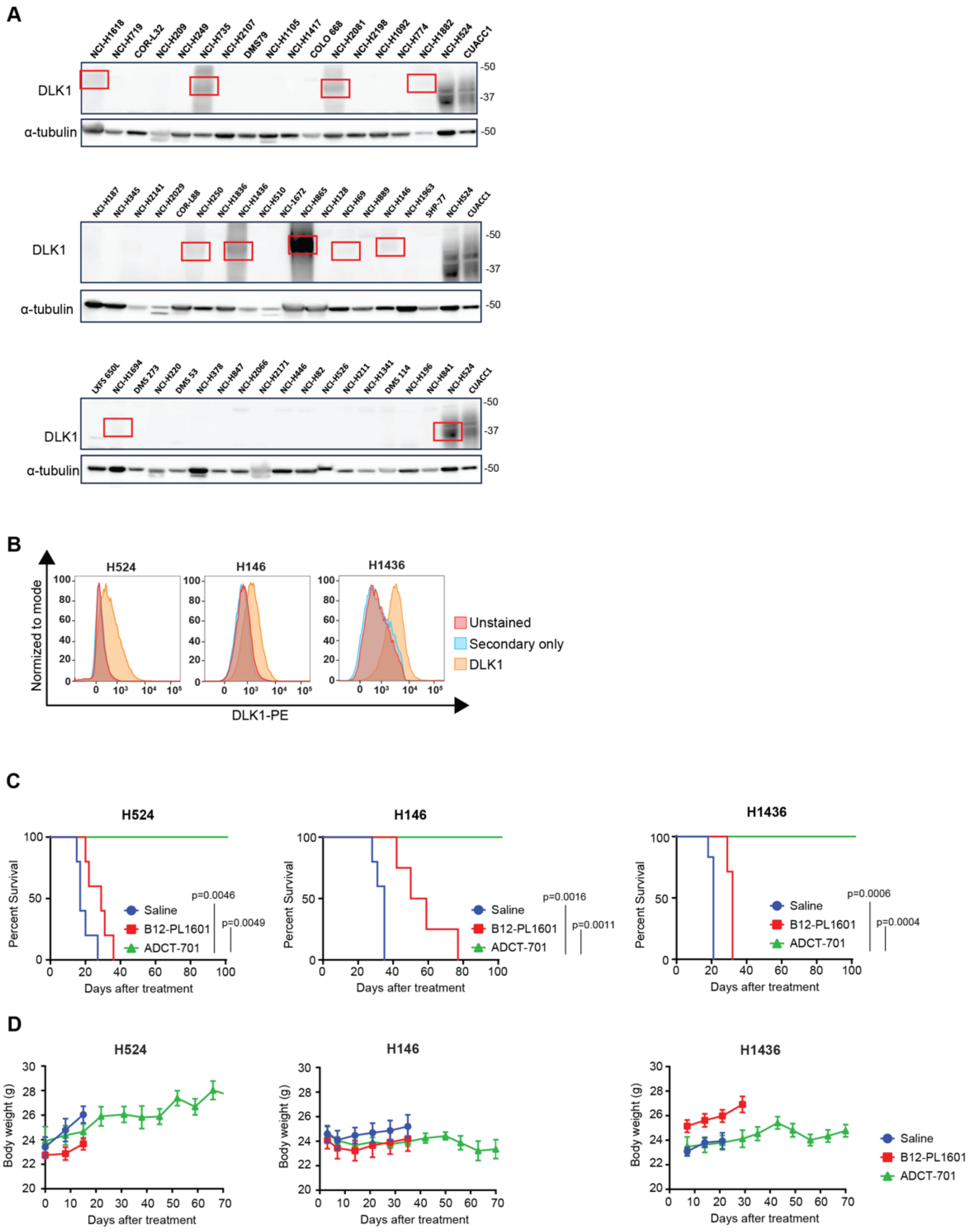

Supplementary Figure 9. Survival and toxicity of ADCT-701 treatment in DLK1^{+} SCLC xenograft models. (A) Immunoblot analysis of DLK1 across 51 SCLC cell lines. Cell lines with positive expression of DLK1 are demarcated by the red rectangles. **(B)** Cell surface DLK1 expression by flow cytometry in 3 SCLC cell lines. **(C)** Survival curves of 3 SCLC xenograft models after treatment with saline, B12-PL1601 or ADCT-701 (1 mg/kg) treatment. **(D)** Body weight curves with saline, B12-PL1601, or ADCT-701 treatment in SCLC xenografts H524, H146, and H1436.

Supplementary Figure 10

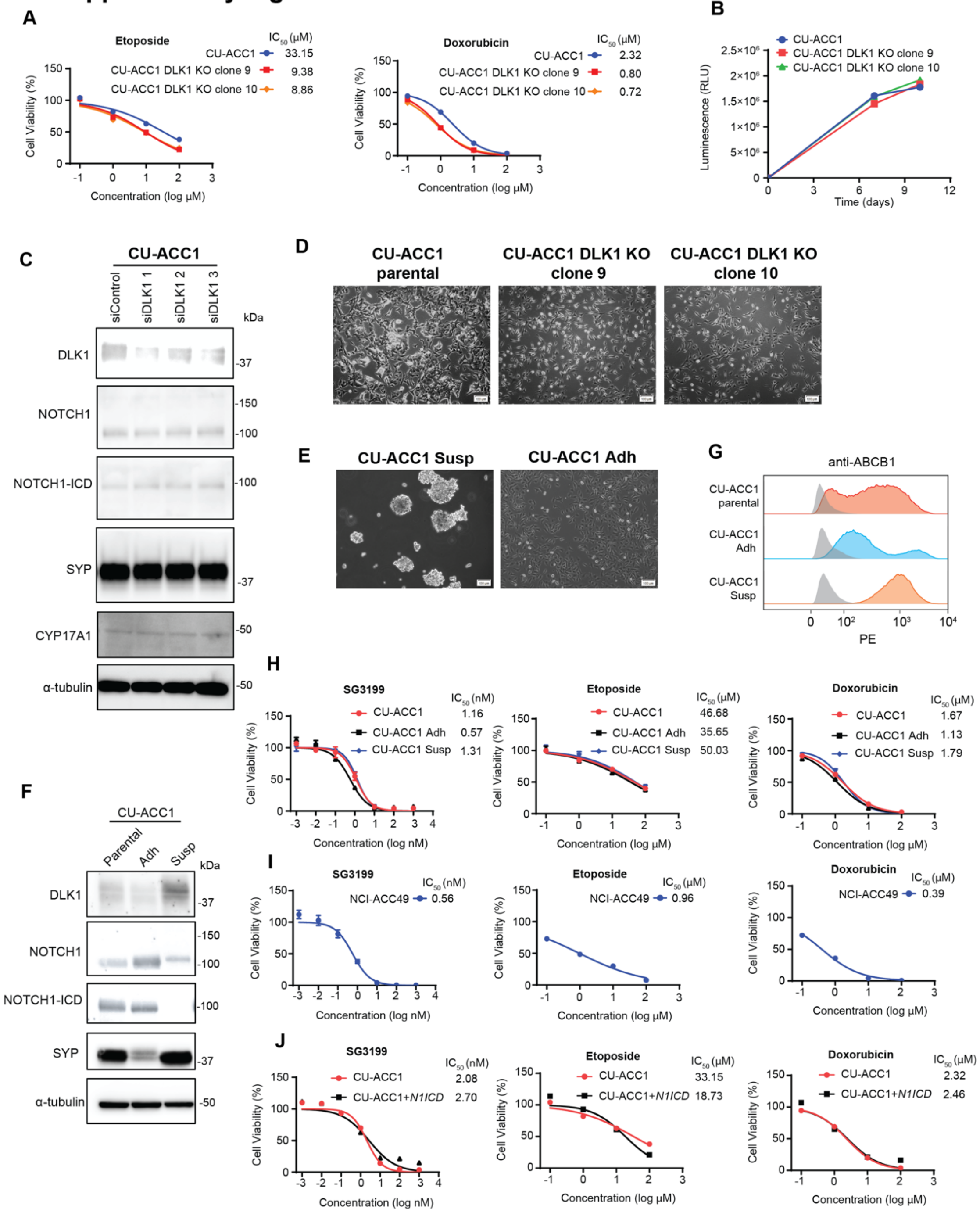

Supplementary Figure 10. Additional data supporting the relationship between DLK1, NOTCH1 signaling, ABCB1, and chemoresistance in ACC. (A) Cytotoxic activity of etoposide and doxorubicin in CU-ACC1 parental and DLK KO clones 9 and 10 cells. **(B)** Proliferation of CU-ACC1 parental and DLK KO clones 9 and 10 cells. **(C)** Immunoblot analysis of DLK1, total NOTCH1 and NOTCH1 intracellular domain (ICD), NE marker synaptophysin (SYP), steroidogenic protein CYP17A1, and loading control (α-tubulin) proteins in siControl compared to siDLK1 in CU-ACC1 cells. **(D)** Photomicrographs of CU-ACC1 parental and DLK KO clones 9 and 10 cells. **(E)** Photomicrographs of CU-ACC1 suspension and adherent cells. **(F)** Immunoblot analysis of DLK1, total NOTCH1 and NOTCH1-ICD, SYP, CYP17A1, and α-tubulin proteins in CU-ACC1 parental, adherent, and suspension cells. **(G)** Flow cytometry histograms of ABCB1 expression in CU-ACC1 parental, adherent, and suspension cells. Shaded gray histograms represent unstained controls for each condition. Cytotoxic activity of SG3199, etoposide, and doxorubicin in **(H)** CU-ACC1 parental, adherent, and suspension cells, **(I)** patient-derived organoid NCI-ACC49, and **(J)** CU-ACC1 cells with and without *N1ICD* overexpression.

Supplementary Figure 11

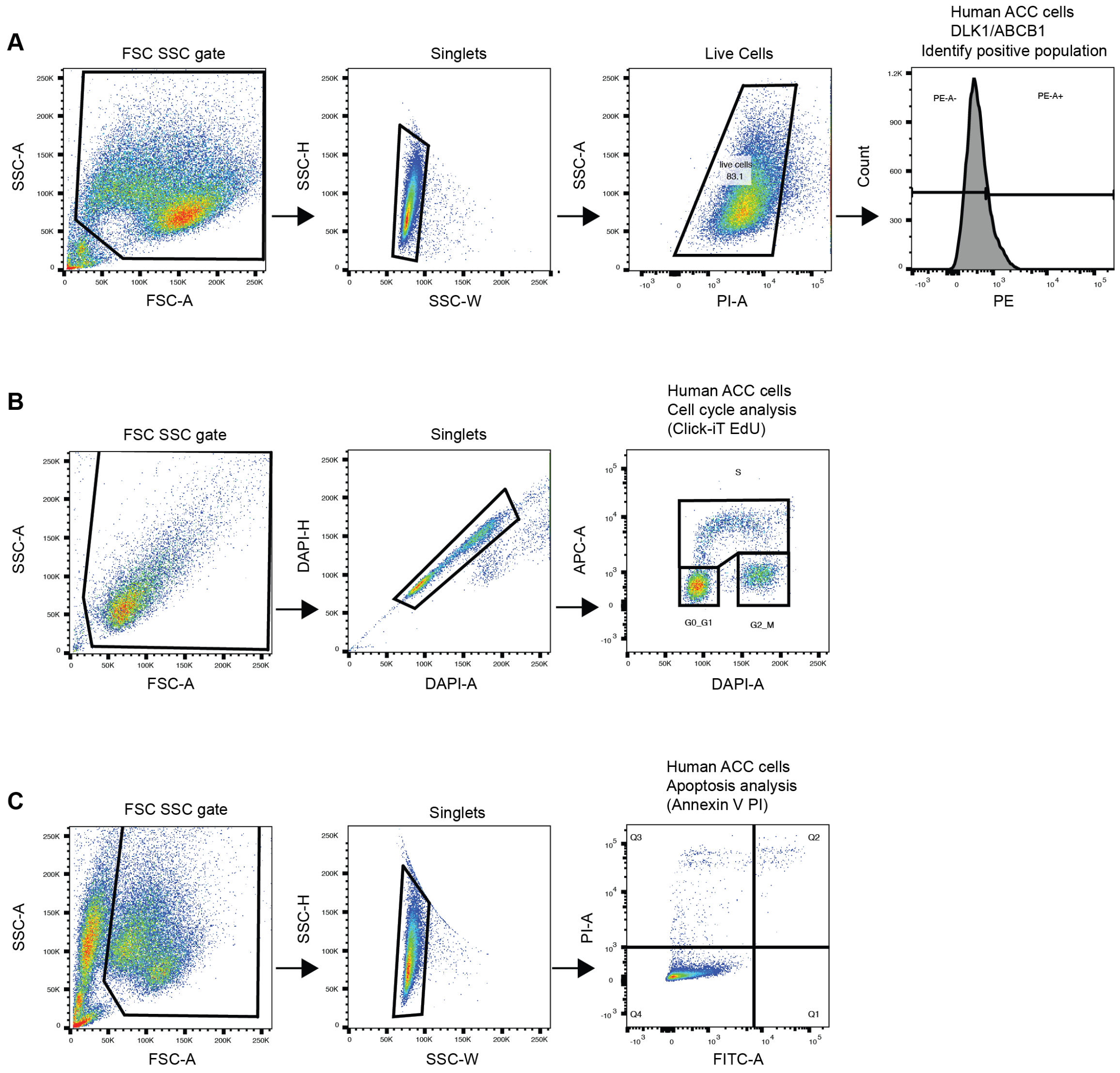

Supplementary Figure 11. Representative flow cytometric gating strategies. (A) For cell surface marker analysis, cells were selected based on FSC/SSC profiles and then further gated for singlets and PI-negative populations. **(B)** For cell cycle analysis, cells were analyzed by APC-EdU and DAPI after single cell selection. **(C)** For apoptosis analysis, cells were analyzed by FITC-Annexin V and PI after single cell selection.
